# Associations between psychological wellbeing, depression, general anxiety, perceived social support, tooth brushing frequency and oral ulcers among adults resident in Nigeria during the first wave of the COVID-19 pandemic

**DOI:** 10.1186/s12903-021-01871-y

**Published:** 2021-10-13

**Authors:** Morenike Oluwatoyin Folayan, Olanrewaju Ibikunle Ibigbami, Ibidunni Olapeju Oloniniyi, Olakunle Oginni, Olutayo Aloba

**Affiliations:** 1 Department of Child Dental Health, Obafemi Awolowo University, Ile-Ife, Nigeria; 2 Department of Mental Health, Obafemi Awolowo University, Ile-Ife, Nigeria

**Keywords:** Oral Health, Mental health, Psychological wellbeing, Adults, Nigeria

## Abstract

**Introduction:**

The aims of this study were to determine the associations between psychological wellbeing, and the frequency of tooth brushing and presence of oral ulcers during the COVID-19 pandemic; and to identify the mediating roles of psychological distress (general anxiety and depression) and perceived social support in the paths of observed associations.

**Methods:**

This cross-sectional study recruited 996 adults in Nigeria between June and August 2020. Data collected through an online survey included outcome variables (decreased frequency of tooth brushing and presence of oral ulcers), explanatory variable (psychological wellbeing), mediators (general anxiety symptoms, depression symptoms and perceived social support) and confounders (age, sex at birth, educational and employment status). Multivariate logistic regression was used to determine the risk indicators for the outcome variables. A path analysis was conducted to identify the indirect effect of mediators on the association between the outcome and explanatory variables.

**Results:**

Of the 966 respondents, 96 (9.9%) reported decreased tooth-brushing frequency and 129 (13.4%) had oral ulcers during the pandemic. The odds of decreased tooth-brushing during the pandemic decreased as the psychological wellbeing increased (AOR: 0.87; 95% CI: 0.83–0.91; *p* < 0.001) and as generalized anxiety symptoms increased (AOR: 0.92; 95% CI: 0.86–0.98; *p* = 0.009). The odds of having an oral ulcer was higher as the generalized anxiety symptoms increased (AOR: 1.15; 95% CI: 01.08–1.21; *p* < 0.001). Only generalized anxiety (indirect effect: 0.02; 95% CI: 0.01–0.04; *P* = 0.014) significantly mediated the relationship between wellbeing and tooth-brushing accounting for approximately 12% of the total effect of wellbeing on decreased toothbrushing. Generalized anxiety (indirect effect 0.05; 95% CI: − 0.07–0.03; *P* < 0.001) also significantly mediated the relationship between wellbeing and presence of oral ulcer accounting for 70% of the total effect of wellbeing on presence of oral ulcer. Depressive symptoms and perceived social support did not significantly mediate the associations between psychological wellbeing, decreased frequency of tooth brushing and the presence of oral ulcers.

**Conclusion:**

Patients who come into the dental clinic with poor oral hygiene or oral ulcers during the COVID-19 pandemic may benefit from screening for generalized anxiety and psychological wellbeing to identify those who will benefit from interventions for mental health challenges.

## Introduction

The first official case of the COVID-19 disease in Nigeria was identified on the 27th of February 2020. At the time of commencement of this study on the 21st of June 2020, the number of COVID-19 cases was 20,244 and the number of deaths was 518. At the time of concluding the data collection on the 6th of August 2020, the number of COVID-19 cases was 45,244 and the number of deaths was 930 [1]. On March 23, 2020, land borders were closed, all international flights were halted, and mandatory institutional quarantine and testing for international returnees to Nigeria were instituted following the confirmation of the initial 30 confirmed cases of COVID-19 in Nigeria [2]. On March 30, 2020, a series of stringent interventions were institutedfor high COVID-19 burden States.These included stay-at-home orders and cessation of non-essential movements and activities at schools and workplaces, bans on religious and social gatherings, cancellation of public events and curfews [2]. These lockdown strategies were eventually extended to the entire country [2, 3].These drastic lockdown measures came with significant economic and social costs for many Nigerians [2, 4]. The pandemic also affected the mental health and wellbeing of people [5] as well as limited the access of many to social support [6].

Wellbeing refers to an individuals’ feeling about their life satisfaction [7]. It incorporates concepts of happiness or social welfare [8]; and is influenced by factors such as access to social support and psychological states [9] such as anxiety and depression which negatively impact self-perceived wellbeing [10]. Nigerians experienced psychological distress such as insomnia, depressive, post-traumatic stress and anxiety symptoms, and suicidal ideation due to the COVID-19 pandemic [11–13]. Many households had their income reduced and access to healthcare services was limited due to the instituted lockdown measures [14]. These psychological and socioeconomic adversities negatively affected the wellbeing of many Nigerians [4].

Psychologicaladversitiessuch as generalized anxiety and depression also had negatively impacts on oral health quality of life [15]. Generalized anxiety and depression are associated with oral ulcers [16] and affect the quality of tooth-brushing [17]. Depression has been shown to have indirect adverse effects on the periodontal health by causing neglect of tooth-brushing due to an unwillingness to take part in physical activities [18, 19]. Depression is also associated with high consumption of refined carbohydrates, use of psychoactive substances, smoking of tobacco, alcoholism and poor dental service utilisation which can increase the likelihood of ulcer-forming oral health conditions [20]. Generally, anxiety is associated with a greater risk for oral ulcers than depression [21].

Poor oral hygiene resulting from poor tooth-brushing will affect the psychological wellbeing of individuals. Poor oral hygiene also affects the general health and is associated with a wide range of medical conditions such as aspiration pneumonia, cardiovascular diseases, stroke, type 2 diabetes, adverse pregnancy outcome [22–24]. Oral ulcers also result in poorer psychological well-being [25]. Poor oral health is associated with poorer lifestyle [26] including social withdrawal, isolation, and low self-esteem thereby setting up a vicious cycle of oral and mental health deterioration [27, 28].

The aims of the study were, therefore, to determine the association between self-reported psychological wellbeing, the frequency of tooth-brushing and the presence of oral ulcers during the COVID-19 pandemic; and to identify if psychological distress (generalized anxiety and depression) and perceived social support partly mediated the observed associations between psychological wellbeing; andthe frequency of tooth-brushing and the presence of oral ulcers. For this study, we recognize the multiple inter-relationships between factors association with oral hygiene and mental health; and that the frequency of tooth-brushing is just one of these factors [29]. We also recognise that beliefs, values, preferences, and thought processes may not always directly connect an intervention like the frequency of tooth-brushing to an outcome like poor oral hygiene [30]. However, considering that few studies have investigated oral health in the context of the COVID-19 pandemic in Nigerian and most developing countries, we used frequency of toothbrushing and oral ulcers as proxies for oral health in the present study [31].

For this study we assessed how the context created by COVID-19 pandemic may have affected an oral health behaviour (tooth-brushing) and its outcomes either directly or indirectly through mental health pathways. We hypothesize that there will be associations between psychological wellbeing, psychological distress (general anxiety and depression), perceived social support, and oral health practice (frequency of tooth-brushing) and oral diseases (oral ulcers) among adults in Nigeria during the COVID-19 pandemic.

## Methods

### Ethical consideration

Ethical approval for the study was obtained from the Research Ethics Committee of the Institute of Public Health, Obafemi Awolowo University Ile-Ife, Osun State Nigeria. The approval number is IPH/OAU/12/1560. Study participants were informed about the study objectives, the inclusion and exclusion criteria, their right to withdraw from study participation and the anonymized nature of the data collection. Participants could only continue with filling the questionnaire if they consented to participating in the study by checking the consent box.

### Study design and study population

The study was a cross-sectional study that recruited adults aged 18 years and older who were resident in Nigeria at the time of their completion of the online survey which was presented as a Google form. The survey was launched from the 21st of June 2020 till the 6th of August 2020. Individuals who were unable to use the internet, or who did not speak English or who had severe cognitive or physical impairments that made it difficult to understand the questions in the survey were excluded from the survey.

### Sample size

Initially, 1045 persons participated in the online survey of whom only 966 (92.4%) had sufficient data for inclusion in analyses. We considered this sample size sufficient as it was larger than similar online surveys that had been carried out in the context of the COVID-19 pandemic which ranged from 500 [32] to 800 [33] and were sufficiently powered to detect significant associations with indicators of wellbeing.

### Study participants’ recruitment

Study participants were recruited through adverts promoted on social media platforms like WhatsApp, Facebook, Instagram and Twitter. These participants were further asked to disseminate the links to those in their own networks using snowball sampling. Web-based modalities for data collection has increased during the COVID-19 pandemic though not without its limitations [34]. Restrictions were applied to the settings so that each participant could only take the survey once. Participants were able to edit their answers freely until they chose to submit. Identifying data were not collected to ensure that responses were anonymous.

### Data collection

#### Outcome variables

*Oral health:* The oral health status of respondents was determined using two questions: did the frequency of tooth-brushing change during the lockdown with the options of ‘Yes (increased)’, ‘Yes (decreased)’ and ‘No’; and did you have oral ulcers during the lockdown with the options Yes or No.

#### Explanatory variables

*Psychological wellbeing assessment:* The World Health Organization-Five (WHO-5) Well-Being Index [35] was used to measure the subjective well-being status of participants. This is a short instrument that consists of five questions scored on a 6-point Likert scale with scores ranging from 0 (none of the time) to 5 (all of the time). The five questions were: (1) ‘I have felt cheerful and in good spirits', (2) ‘I have felt calm and relaxed', (3) ‘I have felt active and vigorous', (4) ‘I woke up feeling fresh and rested' and (5) ‘My daily life has been filled with things that interest me'. The respondents were asked to rate how well each of the statements applied to them within the last 14 days. Possible scores ranged from 0 to 25. The total sum was converted to a percentage by multiplying with four to obtain an aggregate wellbeing index ranging between 0 and 100. Higher scores indicated better wellbeing. The index had been validated with good psychometric properties [36]. The Cronbach's alpha for WHO-5 for this study population was 0.95.

*Generalized anxiety and depressive symptoms assessment*: Depressive and anxiety symptoms were assessed using the Hospital Anxiety and Depression Scale which comprises of 14 questions: seven questions assess anxiety symptoms and the other seven assessed depressive symptoms respectively [37]. Each question was rated on a 4-point Likert scale ranging from 0 (no, not at all) to 3 (yes, definitely). Total scores were derived for anxiety symptoms and depressive symptoms with higher scores indicating more severe symptoms. The scale has been validated for use in Nigeria [38]. For this study, the Cronbach’s alpha for the anxiety and the depression subscales were 0.81 and 0.64 respectively.

*Perceived Social Support*: Perceived social support was measured using the Multidimensional Scale of Perceived Social Support [39]. It consists of 12 questions each rated on a 7-point Likert scale ranging from 1 (very strongly disagree) to 7 (very strongly agree). The total scores, derived by summing the scores on each item, ranged from 12 to 84 with higher scores indicating higher perceived social support. The scale had been validated in Nigeria [40]. For this study, the Cronbach’s alpha was 0.97.

#### Confounders

Information was collected on the age of respondents at last birthday in years, sex at birth, level of highest educational achievement (none, primary, secondary and tertiary) and employment status at the time of data collection (employed, not employed). The study outcome and explanatory variables were assessed with reference to the COVID-19 pandemic period: respondents were asked to self-report on their oral and mental health during the ongoing COVID-19 pandemic period.

### Statistical analyses

Analyses were carried out using the IBM-SPSS software version 26. The variables were described using frequencies and percentages. Tests of association were carried out using Chi-squared tests or student t-tests as appropriate, to determine the associations between the outcome variables (change in frequency of tooth brushing and presence of oral ulcers), the explanatory variables (depression, anxiety, perceive social support and perceived wellbeing) and the confounders (age, sex, educational status and employment status).

Multivariable regression models were used for inferential analysis. Binary logistic regression models were used to tests the associations between each of the explanatory variables and each of the outcome variables while adjusting for confounders. Only variables that showed no collinearity were included in the study model. The estimated coefficients were expressed as adjusted odds ratios (AORs) and their 95% confidence intervals were reported.

Mediation path analyses were conducted with each model consisting of explanatory, mediation, and outcome variables. We used maximum likelihood estimation and estimated means and intercepts. Bootstrapping was used to generate 95% bias-corrected confidence intervals and standard errors using 10,000 bootstraps. Standardized estimates (β) were calculated for direct and indirect effects of anxiety, depression, perceived social support and perceived wellbeing on frequency of tooth-brushing and reports of oral ulcers. The model was considered to have a good fit if the chi square test was not statistically significant: the root mean square error of approximation was < 0.05, the comparative fit index was > 0.09 and the Tucker- Lewis index was > 0.09. Significance was set at the 5% level. Statistical analyses were conducted with SPSS version 22.0 and mediation analyses including the direct, indirect and total effects were estimated with PROCESS procedure for SPSS version 3.5.3 [41].

## Results

Table 1 highlights the factors associated with the frequency of tooth-brushing and oral ulcers during the pandemic. Of the 966 respondents, 96 (9.9%) reported a decrease in tooth-brushing frequency and 129 (13.4%) had oral ulcers during the pandemic. More respondents who had low psychological wellbeing (*p* < 0.001), who were younger (*p* = 0.015) and who had more severe depressive symptoms decreased their frequency of their tooth brushing during the pandemic (*p* = 0.032). Also, more male than female respondents (*p* = 0.005), older respondents (*p* = 0.021), those with tertiary education (*p* < 0.001), those with worse psychological wellbeing (*p* = 0.001), more severe general anxiety symptoms (*p* < 0.001), more severe depressive symptoms (*p* < 0.001) and lower perceived social support (*p* = 0.035) had oral ulcers during the pandemic.

**Table 1:** Profile of tooth brushing frequency of tooth brushing and oral ulcers among adults resident in Nigeria during the COVID-19 pandemic (N = 966)

Variables	Total	Decreased tooth brushing frequency		*p*-value	Oral ulcers		*p*-value
		Yes	No		Yes	No	
		N = 94	N = 872		N = 129	N = 837	
*Sex*							
Male	487 (50.41)	40 (42.55)	447 (51.26)	0.109	80 (62.02)	407 (42.13)	0.005
Female	479 (49.59)	54 (57.45)	425 (48.74)		49 (37.98)	430 (44.52)	
*Age*							
Mean (standard deviation)	31.25 (9.90)	28.89 (9.76)	31.51 (9.88)	0.015	33.12 (12.30)	30.96 (9.44)	0.021
*Educational status*							
None	74 (7.66)	5 (5.32)	69 (7.91)	0.390	15 (11.63)	59 (6.11)	< 0.001
Primary	21 (2.17)	4 (4.26)	17 (1.95)		8 (6.20)	13 (1.35)	
Secondary	110 (11.39)	12 (12.77)	98 (11.24)		21 (16.28)	89 (9.21)	
Tertiary	761 (78.78)	73 (77.66)	688 (78.90)		85 (65.89)	676 (69.98)	
*Employment status*							
Employed	562 (58.18)	48 (51.06)	514 (58.94)	0.141	67 (51.94)	495 (51.24)	0.123
Not employed	404 (41.82)	46 (48.84)	358 (41.06)		62 (48.06)	342 (35.40)	
*Psychological wellbeing*	20.56 (5.64)	16.81 (6.16)	20.96 (5.43)	< 0.001	19.07 (5.28)	20.79 (5.66)	0.001
*General anxiety symptoms*	15.82 (4.50)	15.79 (4.47)	15.82 (4.51)	0.943	18.11 (3.72)	15.47 (4.51)	< 0.001
*Depressive symptoms*	14.80 (3.55)	15.54 (4.08)	14.71 (3.48)	0.032	15.82 (2.89)	14.64 (3.62)	< 0.001
*Perceived social support*	36.76 (17.30)	38.75 (12.23)	36.55 (17.75)	0.243	33.78 (17.82)	37.22 (17.18)	0.035

Table 2 highlights the risk indicators for decrease in tooth-brushing frequency and the presence of oral ulcers during the COVID-19 pandemic. The two factors significantly associated with a decrease in tooth-brushing were psychological wellbeing, and general anxiety symptoms. The odds of decreased frequency of tooth-brushing during the pandemic decreased as the psychological wellbeing increased (AOR: 0.87; 95% CI: 0.83–0.91; *p* < 0.001) and as the severity of general anxiety symptoms increased (AOR: 0.92; 95% CI: 0.86–0.98; *p* = 0.009).

**Table 2:** Multivariate regression of factors associated with decreased frequency of tooth brushing and presence of oral ulcers among adults resident in Nigeria during the COVID-19 pandemic (N = 966)

Variables	Decreased tooth-brushing frequency		Presence of oral ulcers	
	AOR (95% CI)	*p* value	AOR (95% CI)	*p* value
*Sex*				
Male	0.91 (0.57–1.45)	0.679	0.57 (0.37–0.86)	0.008
Female	1.00	–	1.00	**–**
*Age*				
Mean (standard deviation)	0.98 (0.95–1.01)	0.195	1.031 (1.01–1.05)	0.003
*Educational status*				
None	1.00	–	1.00	–
Primary	3.22 (0.73–14.13)	0.121	1.34 (0.70–2.57)	0.375
Secondary	0.84 (0.25–2.73)	0.769	3.88 (1.47–10.14)	0.006
Tertiary	0.91 (0.34–2.44)	0.853	2.31 (1.28–4.17)	0.005
*Employment status*				
Employed	0.97 (0.57–1.65)	0.896	1.38 (0.46–1.13)	0.157
Not employed	1.00	–	1.00	–
*Psychological wellbeing*	0.87 (0.83–0.91)	< 0.001	0.97 (0.94–1.01)	0.174
*General anxiety symptoms*	0.92 (0.86–0.98)	0.009	1.15 (1.08–1.21)	< 0.001
*Depressive symptoms*	1.03 (0.95–1.12)	0.440	1.02 (0.94–1.20)	0.665
*Perceived social support*	1.02 (0.99–1.03)	0.075	1.01 (0.99–1.02)	0.109

Factors significantly associated with having oral ulcers during the pandemic were general anxiety symptoms, sex at birth, age and educational status. The odds of having an oral ulcer was higher as the general anxiety symptoms increased in severity (AOR: 1.15; 95% CI: 01.08–1.21; *p* < 0.001); as the age increased (AOR: 1.031; 95% CI: 1.01–1.05; *p* = 0.003); and for those with secondary (AOR: 3.88; 95% CI: 1.47–10.14; *p* = 0.006) and tertiary (AOR: 2.31; 95% CI: 1.28–4.17; *p* = 0.005) education when compared with respondents with no education. The odds of having oral ulcer was lower for men when compared to women (AOR: 0.57; 95% CI: 0.37–0.86; *p* = 0.008).

**Fig. 1:**
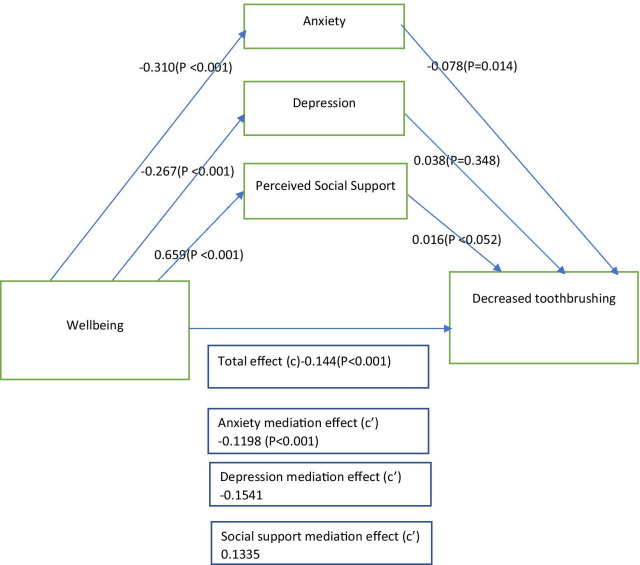
Mediational analysis model showing the indirect effect of general anxiety, depressive symptoms on the association between wellbeing and decreased toothbrushing

As shown in Fig. 1, depressive symptoms (β: − 0.01; 95% CI: 0.03–0.01) and perceived social support (β: 0.01; 95% CI: 0.001–0.02) had no significant mediator effect on the relationship between wellbeing and decreased toothbrushing. We noted that anxiety symptoms, after being introduced as a mediator significantly (indirect effect: β: 0.02; 95% CI: 0.01–0.04; *P* = 0.014) reduced the effect of wellbeing on the dependent variable (frequency of toothbrushing), accounting for approximately 12% of the total effect of wellbeing on decreased toothbrushing.

**Fig. 2:**
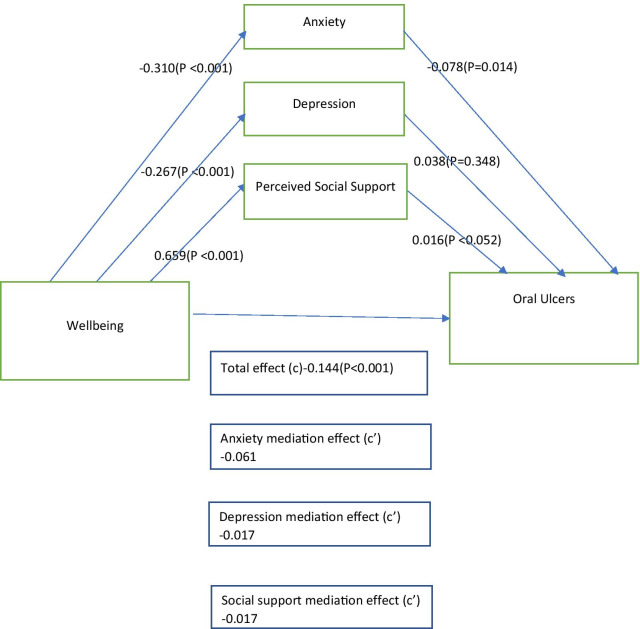
Mediational analysis model showing the indirect effect of general anxiety, depressive symptoms on the association between wellbeing and oral ulcer

As shown in Fig. 2, we also found that depressive symptoms (β: − 0.002; 95% CI: − 0.02–0.02) and perceived social support (β: 0.002; 95% CI: − 0.006–0.010) did not significantly mediate the relationship between wellbeing and oral ulcer. Anxiety symptoms, after being introduced was the only significant mediator (indirect effect: β 0.05; 95% CI: − 0.07–0.03; *P* < 0.001) between wellbeing and oral ulcers, accounting for 70% of the total effect of wellbeing on presence of oral ulcers.

## Discussion

The study provides evidence that about 10% of respondents reported decreased frequency of tooth-brushing and about 13% of respondents had oral ulcers during the COVID-19 pandemic in Nigeria. Worsening psychological wellbeing and increased severity of generalized anxiety symptoms were associated with decreased frequency of tooth-brushing while increased severity of generalized anxiety was associated with the presence of oral ulcers. Generalized anxiety also partly mediated the association between psychological wellbeing and decreased frequency of tooth brushing and presence of oral ulcers. The mediation effect of generalized anxiety was greater for the association between psychological wellbeing and oral ulcers than that for the association between psychological wellbeing and decreased frequency of tooth-brushing. Depressive symptoms and perceived social support were not associated with decreased frequency of tooth-brushing and presence of oral ulcers in adults during the COVID-19 pandemic in Nigeria.

One of the strengths of the study is the empirical evidence provided on the impact of the COVID-19 pandemic on the oral health of individuals. Prior studies had shown that SARS-CoV-2 infection is a risk factor for ageusia. There are case reports of COVID-19 being associated with necrotizing periodontal disease, oral ulcers, blisters, salivary gland alterations, white and erythematous plaques, and recurrent herpetic lesions [42–44]. The suggested pathways for these COVID-19 related oral lesions was mediation by SARS-CoV-2-associated tropism of endothelial cells and COVID-19-mediated endothelitis that promotes inflammation of oral tissues [45]. Our study suggests that oral ulcers may result from psychological health mediated pathways; specifically, poor wellbeing was associated with generalized anxiety symptoms which were in turn associated with decreased frequency of tooth-brushing and increased oral ulcers. Poor oral hygiene resulting from decreased tooth-brushing is a predisposing factor for necrotizing periodontal disease [46]. The COVID-19 pandemic was associated with food insecurity, psychological stress, malnutrition, and poor sleeping habit [47, 48]; factors that increase the risk for oral ulcer-causing lesions like necrotising periodontal disease [49].

Our study, however, has several limitations. First, it was a cross-sectional study and so we cannot infer cause-effect relationships between the variables. For example, in addition to the pathways investigated in the present study, it is possible that oral health conditions also lead to decreased wellbeing. However, this possibility would be better investigated in longitudinal designs. Secondly, the data was collected using an online survey which may have excluded eligible participants who could not access the internet and this may limit the generalizability of the study findings. Thirdly, we did not explore changes in the diet which could also contribute to changes in oral health. Also, we did not rule out SARS-CoV-2 infection, tobacco use and other underlying medical problems that could be alternative causes of oral ulcers. Fourthly, there is also the risk for social desirability bias associated with self-reported questionnaires which may result in the under-reporting of mental health status and over-reporting of toothbrushing frequency. There is also the possibility of data contamination resulting from multiple individuals from the same household filling the questionnaire. Finally, the data on oral ulcer has an implicit assumption that the data collected was related to stress ulcers. There are other forms of ulcers that may have developed that are not related to stress thereby leading to a possible overestimation of the ulcers resulting directly or indirectly from the pandemic. Despite these limitations, we provide new evidence that suggests that the COVID-19 pandemic may be associated with changes in the oral health and oral health habits through direct and indirect associations with mental health.

Prior studies have indicated that better psychological wellbeing mediates better oral health quality of life [50]; and that mental health problems are directly and indirectly associated with poor oral health [15–19]. The findings of this study not only corroborate prior findings on the impact of mental health on oral health; but alsoindicate that these impacts are both direct and indirect during the COVID-19 pandemic.

The relationships between psychological wellbeing, generalized anxiety, frequency of tooth brushing, and oral ulcer appears complex. Prior studies indicate that generalized anxiety and depression were heightened during the pandemic globally [51] and in Nigeria [32]. The negative impacts of generalized anxiety and depression on psychological wellbeing has been previously reported [52, 53]; as have the negative impact of reduced psychological wellbeing, depression and generalized anxiety on oral health [15–20]; and the positive impact of access to social support on psychological wellbeing, depression, generalized anxiety and oral health [54]. These relationships may however be altered during a health crisis like the COVID-19 pandemic. We found that generalized anxiety (but not depression and perceived social support) wasassociated with the presence of oral ulcers and decreased frequency of tooth brushing. Generalized anxiety may cause an elevation in salivary cortisol and/or in immune regulatory activity in inflammation by increasing the quantity and activity of leukocytes thereby increasing the likelihood of oral ulcers. Generalized anxiety may also be associated with cheek or lip biting, or other actions causing injury to the oral mucosa [55].Although we found no literature indicating that pandemics may alter the pathway by which mental health problems may be associated with diseases, our findings indicate this possibility and the need for further investigation.

Poor psychological wellbeing had been associated with multiple health risk habits [56]. We did not come across any study indicating an association between psychological wellbeing and toothbrushing, however, our finding suggests that a decrease in the frequency of tooth-brushing may also reflect poor psychological wellbeing. Screening for poor psychological wellbeing in adults who present to a dental clinic with histories decreased frequency of tooth-brushing during stressful periods like the COVID-19 pandemic may help ensure prompt mental health support is provided when such needs are identified. It may also be important to manage the underlying mental health difficulties to improve oral health habits such as tooth-brushing and ensure prompt healing of oral ulcers. Normal tooth-brushing frequency also needs to be restored to reduce the risk for poor oral hygiene; a known risk factor for general health problems [57].

Consistent with a prior study which indicated that generalized anxiety had a stronger association with oral ulcers when compared with depression [27], our inability to identify a significant mediation effect for depressive symptoms and perceived social support in the relationship between psychological wellbeing and decreased frequency of tooth brushing may suggest that anxiety symptoms are more salient mental health correlates of oral health. This would be consistent with findings from separate analyses of the present dataset in which more COVID-19-related stressors were associated with generalized anxiety symptoms compared to depressive symptom [58].

## Conclusion

Poor psychological wellbeing associated with the COVID-19 pandemic may be in turn associated withdecreased frequency of tooth-brushing. Generalized anxiety resulting from the COVID-19 pandemic may also be associated with decreased frequency of tooth-brushing and oral ulcers. Generalized anxiety partly explains the relationship between psychological wellbeing and decreased frequency of tooth brushing. However, we found no direct or indirect effects for depressive symptoms and perceived social support in the associations between psychosocial wellbeing, frequency of tooth-brushing and presence of oral ulcers during the COVID-19 pandemic. Patients who present at dental clinics during the COVID-19 pandemic may need to be screened for generalized anxiety and poor psychological wellbeing; and may need to be managed alongside mental health professionals.

## Data Availability

All data generated for this study are presented in the manuscript. The dataset for the online study data can however be accessible on reasonable request from one the study authors, Olakunle Oginni.

## Abbreviations

β: Coefficient
CI: Confidence interval
SARS-CoV-2: Severe Acute Respiratory Syndrome Corona Virus 2
COVID-19: Corona Virus Disease 2019
